# GLOBAL DEMOGRAPHIC-CULTURAL SHIFTS AND ‘KINLESSNESS’: RECONSIDERING INTERGENERATIONAL THEORIES

**DOI:** 10.1093/geroni/igae098.4201

**Published:** 2024-12-31

**Authors:** Christine Mair, Rose Anderson

**Affiliations:** University of Maryland Baltimore County, Baltimore, Maryland, United States; University of Maryland Baltimore, Baltimore, Maryland, United States

## Abstract

Demographic and cultural shifts such as declining fertility, expanding longevity, and shifts toward individualism (e.g., living alone) have contributed to changes in intergenerational relationships among aging populations cross-nationally. Specifically, the proportion of older adults who do not have children or partners (“kinless”) is expected to increase substantially in nearly all regions of the globe over the next 50 years. Yet, these shifts do not occur evenly across low-, middle-, and high-income countries. Such substantial demographic-cultural changes and cross-national inequalities will require that scholars reconsider existing concepts and theories of intergenerational relationships. In this paper, we analyze publicly available aggregate, country-level data from Our World in Data and the World Values Survey from multiple time periods to explore trends in fertility, longevity, population age composition, rates of living alone and one-person households, rates of “kinlessness”, and cultural endorsement of values associated with individualism and friendship in low-, middle-, and high-income countries. The results illustrate differences by countries and regions, which highlight areas for future conceptual development. Based on these results, we propose new directions for gerontological theories, measurement, and policy regarding intergenerational relationships, parenthood and partnership, childlessness and “kinlessness,” and siblings, extended family, and friendships that address the shifting characteristics of intergenerational relationships globally.

